# Psychosocial Impact of Assistive Devices and Other Technologies on Deaf and Hard of Hearing People

**DOI:** 10.3390/ijerph18147259

**Published:** 2021-07-07

**Authors:** Estíbaliz Jiménez-Arberas, Emiliano Díez

**Affiliations:** 1 Faculty Padre Ossó (Asociated Center of the University of Oviedo), Degree in Occupational Therapy, University of Oviedo, 33008 Oviedo, Spain; estibaliz@facultadpadreosso.es; 2 Institute for Community Inclusion (INICO), University of Salamanca, 37005 Salamanca, Spain

**Keywords:** assistive technology, hearing aids, outcomes

## Abstract

Deaf and hard of hearing people use a variety of assistive devices and technologies as a strategy to mitigate, counter or compensate for life difficulties resulting from hearing loss. Although outcome measures are commonly used with hearing aids, few studies have explored the perceived psychosocial impact of other assistive devices and technologies or the factors leading to their abandonment or lack of use. Therefore, the main aim of this study was to assess the psychosocial impact of different assistive devices on deaf and hard of hearing people using the Psychosocial Impact of Assistive Devices Scale. The sample was made up of 291 individuals, 176 women and 115 men, with an average age of 56.12 years (standard deviation (SD) = 25.11), who were all users of different assistive devices. Overall, the results of the study showed that the use of assistive devices had a positive psychosocial impact, although this impact varied slightly depending on the specific type of device. Moreover, a relationship was identified between the psychosocial impact and the probability of future abandonment of a hearing aid or a cochlear implant. The results point to the importance of considering the psychosocial impact derived from the use of a device as a relevant variable in the adoption process of assistive technologies for deaf and hard of hearing people.

## 1. Introduction

Hearing loss is a global issue and one of the main concerns of the World Health Organisation (WHO) [1] which estimates that there will be nearly 2.5 billion people with some degree of hearing problems in 2050. Currently, the WHO estimates that there are 433 million people with disabling hearing loss around the world (5% of the global population). Many of these people use assistive devices and technologies to help them hear. Nevertheless, the World Report on Hearing [2] indicates that only 17% of the population that would benefit from using a hearing aid (HA) uses it. This gap in non-use ranges from 77% in Europe to 90% in Africa. Specifically, the most commonly used devices for hearing loss are HAs (in-the-ear, behind-the-ear, in-the-canal or completely-in-canal) [3,4] and cochlear implants [5]. In addition, other assistive technologies are frequently used like video relay services (VRS) [6], mobile phones [7] (mobile applications such as messaging or video call: Telegram, WhatsApp, Texmee, etc.) or Internet video telephony systems [8,9].

Given the high frequency of use of all these technologies, one of the most active fields of research in audiological rehabilitation is dedicated to outcome measures derived from the use of assistive technologies (AT) for audition [10]. Broadly speaking, there are general instruments for measuring the outcomes of AT, which are applicable to any form of AT and based on general concepts (such as psychosocial impact), and specific instruments for people with auditory disabilities to assess specific aspects of their interaction with a particular type of AT and other information and communication technologies (ICT). For example, some commonly used specific instruments are the Abbreviated Profile of Hearing Aid Benefit (APHAB) [11], which aims to document the outcome of a hearing aid fitting, and the Measuring Satisfaction with Amplification in Daily Life scale (SADL) [12] to identify users’ levels of knowledge of AT for amplifying sound and for assisting hearing in general. Among the general instruments available are the Psychosocial Impact of Assistive Devices Scale (PIADS) [13], which assesses users’ subjective perceptions of the changes that occur when they adopt an assistive device. As well as having good psychometric properties, the PIADS scale has been used to measure the impact of different forms of AT and ICT, including devices specifically designed for the deaf and hard of hearing [14,15].

In the field of outcome measures, it is important to conduct studies to design assessment tools with good metric properties and to evaluate outcomes not only in terms of hearing improvement, but also in relation to their impact on psychosocial well-being and occupational performance [16,17,18,19]. The improvement of services for the provision of assistive technologies is a major challenge [20] and having adequate models with the corresponding assessment tools that help to take decisions and evaluate outcomes in interventions with assistive devices is a priority in this area.

There is a significant body of research examining the performance and usefulness of different generic and specific outcome measures for hearing aids. For example, Saunders and Jutai [15] examined the PIADS scale and specific instruments such as the APHAB (Abbreviated Profile of Hearing Aid Benefit) [11], the ECHO questionnaire (Expected Consequences of Hearing Aid Ownership [21]) and the SADL scale (Satisfaction with Amplification in Daily Life [12]). They concluded that the PIADS scale could be a useful addition to the specific outcome measures for hearing aids, especially for comparing the functional and psychosocial outcomes of different devices. Moreover, Saunders and Jutai note that the benefits of using AT are linked to a greater duration of use. In a similar vein, previous studies have demonstrated differences in the impact based on variables as the frequency of use (users who used more frequently HA had a greater positive impact in PIADS scale) [22], the context of use (environments where they carry out their activities of daily life: family context, work context, community context etc.), and the type of daily life and social activities in which the products are used (if the AT user is active and has family networks, friends, or other social activities, they will be more successful in using HA) [23]. With regard to other assistive devices, Chen et al. [24] carried out a study in which they evaluated the psychosocial impact of cochlear implants (CI), confirming their positive impact on both users and carers.

More generally, a number of studies have assessed different aspects relating to the use of AT in deaf and hard of hearing, including the psychosocial impact deriving from its use [14,25,26,27], lack of use of AT due to psychosocial factors [28], attitudes [29,30], stigma [31] and quality of life based on levels of use [14,32]. Other studies research specific assistive devices and other technologies for Deaf and their psychosocial impact, such as VRS use and hearing aids [15,25,33,34].

Another interesting area in the field of outcome measures is the study of the abandonment of AT. Despite the usefulness of assistive technologies, in some cases adopted devices are abandoned; in the case of hearing aids, studies have reported abandonment rates of up to 78% [35]. The factors leading to abandonment include those related to the device itself, such as malfunctions [36,37] or aesthetic concerns [38], and those relating to the characteristics of the user, such as the degree of hearing loss [39] or the stigma attached to the use of AT [40]. Meanwhile, psychosocial factors such as self-esteem and extroversion/introversion appear to be linked to success/failure [41,42]. Other relevant factors include ineffective funding mechanisms for acquiring and maintaining AT [38,43].

Despite the extensive body of literature on outcome measures in auditory assistive devices, very few studies have focused on evaluating their psychosocial impact. Among these, fewer still have analysed the perceived impact of the use of different devices in a differential manner. Therefore, this study aims to evaluate the psychosocial impact deriving from the use of different AT for communication by deaf and hard of hearing people, as well as to analyse the psychosocial impact of different types of device and the effect of these differences on the abandonment or lack of use of AT.

## 2. Materials and Methods

### 2.1. Participants

The sample for this study was selected using non-probability convenience sampling. The sample was made up of 291 individuals, 176 women and 115 men, with an average age of 56.12 years (SD = 25.11). The AT covered by the study were CI (n = 30), hearing aids (n = 137) and ICT (n = 124), including mobile devices (e.g., messaging mobile applications such as Telegram, WhatsApp or Texmee) (n = 26), VRS (n = 66) or software to conduct video-calls like ooVoo (n = 17), a cross-platform instant voice and text messaging app, and Skype (n = 15). The study was carried out with users of associations for deaf and hard of hearing people at residential centres in several different regions in Spain. A total of 33 centres were contacted, of which 16 participated in the study (see Table 1). The inclusion criteria for the study were as follows: participants should be aged over 18, with a hearing disability and no other additional disability (those with blindness or deafblindness such as Usher syndrome were excluded), and they had to use some kind of AT or ICT (participants had to use a single device to mitigate, compensate or neutralize partial or total hearing loss). All participants received detailed information about the nature of the study and signed an informed consent form.

### 2.2. Procedure

The data collection procedure was based on the application of the researchers’ custom-made socio-demographic questionnaire and the PIADS scale. In this study, data collection was carried out by means of individual interviews, lasting between 30 and 90 min, depending on the characteristics of the participants. Questions were supplied in writing and in Spanish sign language. Contact was made through in-person and/or virtual interviews at different associations with professionals working directly with hard of hearing and/or deaf people. The study was structured in three phases; the first phase was aimed to obtain data from deaf and hard of hearing individuals who used ICTs to improve daily occupational performance, especially in areas such as the instrumental activities of daily life such as communication management, use of financial management, maintain security and respond to emergency (e.g., mobile phone apps like WhatsApp, Telegram, ooVoo or other computer software, like Skype, etc.). Participants from this group answered by means of a self-administered online questionnaire. The second phase was aimed at obtaining data from people who only used hearing aids (different types and models such as: spectacle, deep-insertion, in-the-canal, behind-the-ear, etc.). For this group, the interviews were carried out in person face to face. Finally, the third phase involved obtaining data from individuals who used CI exclusively (they only used one CI and not a CI and hearing aid simultaneously), by way of face to face interviews. Detailed information about the study and its objectives was provided and the researchers requested collaboration from the associations and centres, as well as from the professionals. If they accepted, several dates were proposed for conducting the interviews and the administration of questionnaires. Following the initial evaluation, the participants using hearing aids and cochlear implants were contacted again 12 months later to find out whether they continued to use the same AT or whether they had discontinued their use (it was not possible to contact users of information and communication technologies group as contact was mostly virtual). This contact was made directly with participants or, where necessary, with their associations or centres. One hearing aid user could not be evaluated because he had passed away.

### 2.3. Instruments

#### 2.3.1. Psychosocial Impact of Assistive Devices Scale (PIADS)

The PIADS scale is a 26-item self-report survey which assesses the functional independence, wellbeing and quality of life linked to the use of assistive technology [44]. It is a reliable and valid measure, which can be applied to a wide range of support products and to different types of disability and health status [43,45]. The scores in the PIADS are divided into three subscales: (1) competency, which reflects perceptions of functional capacity, independence and performance; (2) adaptability, which reflects inclination or motivation to participate socially and take risks; and (3) self-esteem, which reflects confidence, self-esteem and emotional wellbeing. The PIADS scale requires respondents to assess how a specific assistive device affects their lives and makes them feel. In order to do this, they must respond to all items using a 7-point scale which extends from −3 (it has reduced) to +3 (it has increased). The middle point, zero, would indicate that no impact or change has been perceived to result from the use of the device. In this study, the adapted Spanish version by Quinteiro [46] was used. The PIADS scale has been used in AT for the deaf and hard of hearing, such as hearing aids [47] and VRS [14].

#### 2.3.2. Matching Person and Technology (MPT)

The Survey of Technology Use-Consumer (SOTU-C), part of the matching person and technology (MPT) model [48], is a form designed to assess the quality of the relationship that the user has with technologies. SOTU-C examines the influences that have a greater impact on the use or non-use, by the person, of AT in general. SOTU-C was used to explore the general predisposition of deaf and hard of hearing people to new technologies. The instrument employs a semantic differential scale with 3 categories (positive, neutral and negative) to examine the most frequently used technologies, the global experiences with those technologies, opinions on the use of new devices, typical activities and personal/social characteristics of assistive device users. The score is obtained with the total number of positive, neutral and negative responses in each category. If the total positive responses outweigh the negative ones, then it can be said that the user has a favorable predisposition towards the use of technology.

## 3. Results

In order to attain the study objectives, several analyses of the data were conducted.

Firstly, a descriptive analysis of the total PIADS scale and its three subscales was carried out. This analysis showed that the average impact was positive for the three subscales of self-esteem (M = 1.15; SD = 1.12), adaptability (M = 1.37; SD = 1.1) and competence (M = 1.23; SD = 1), and for the total PIADS scale (M = 1.24; SD = 0.99).

Secondly, the scores for the total scale and the three subscales were calculated grouping each type of assistive device according to four groups: HA, CI, ICT and VRS. As shown in Table 2, globally, positive values were obtained for all groups of assistive devices. To further explore possible differences in the impact as a function of the type of device, and taking into account that normality and homocedasticity assumptions were violated for most of the groups involved in the analysis, a set of robust analyses of variance (ANOVAs, trimmed means, level 0.2, walrus R package) were performed.

First, and in order to determine whether the type of loss could have a significant effect on psychosocial impact, a robust two-way ANOVA with type of hearing loss (hypoacusis or cophosis) and type-of-device as between variables, and PIADS global score as dependent variable was performed. Results showed significant main effects of type of device [Q = 20.17, *p* < 0.01] and type of hearing loss [Q = 5.07, *p* < 0.05] but, importantly, a non-significant interaction [Q = 1.05, *p* = 0.80]. Similar analysis showed non-significant interactions for age [Q = 2.56, *p* = 0.51], gender [Q = 3.39, *p* = 0.36] or onset of loss [Q = 0.14, *p* = 0.99]. Therefore, neither of those variables were included in the subsequent analyses.

Second, a robust two-way mixed ANOVA with type-of-device as between variable and PIADS subdimensions scores as a within variable showed significant effects for type-of-device [F (3, 92) = 10.08, *p* < 0.001], PIADS scores [F (2, 123) = 6.73, *p* < 0.01] and the interaction [F (6, 86) = 5.20, *p* < 0.001]. To explore those main effects and the interaction a set of robust one factor ANOVAs were performed for global PIADS score and for each subdimension score.

For PIADS global score, the analysis showed differences as a function of the type-of-device groups [F = 12.16, *p* < 0.001]. Post-hoc tests showed a significantly lower psychosocial impact for ICTs compared to hearing aids [Psi-hat = 0.81 (0.40, 1.22); *p* < 0.001], CIs [Psi-hat = 1.02 (0.18, 1.85); *p* < 0.01] and VRSs [Psi-hat = −0.78 (−1.24, −0.32); *p* < 0.001]. No other differences were found to be significant.

A quite similar pattern of results was obtained for subdimensions of competence [F = 11.31, *p* < 0.001] and self-esteem [F = 12.86, *p* < 0.001]. In relation to competence, post-hoc tests showed lower psychosocial impact for ICTs in comparison to hearing aids [Psi-hat = 0.82 (0.37, 1.27); *p* < 0.001], CIs [Psi-hat = 1.02 (0.11, 1.92); *p* < 0.05] and VRSs [Psi-hat = −0.94 (−1.44, −0.45); *p* < 0.001]. The same results were found for self-esteem dimension, with lower impact scores for ICTs in comparison to hearing aids [Psi-hat = 1.03 (0.57, 1.49); *p* < 0.001], CIs [Psi-hat = 1.09 (0.16, 2.02); *p* < 0.05] and VRSs [Psi-hat = −0.56 (−1.06, −0.07); *p* < 0.05]. For adaptability dimension [F = 4.81, *p* < 0.01], post-hoc tests showed lower scores for ICTs in comparison to hearing aids [Psi-hat = 0.53 (0.03, 1.04); *p* < 0.05] and VRSs [Psi-hat = −0.79 (−1.43, −0.16); *p* < 0.01].

Finally, the analysis also examined abandonment of AT. To this end, the researchers contacted the participants who used hearing aids and CI one year after the first round of data collection for the study and asked whether or not they continued to use the AT. A total of 25 participants had abandoned hearing aids and six participants had abandoned cochlear implants. The results are shown in Table 3 below.

**Figure 1:**
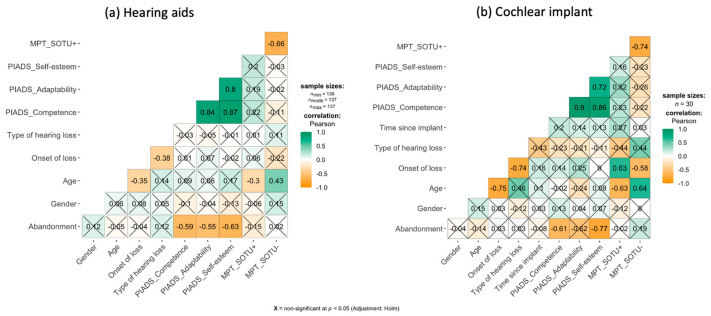
Correlogram showing correlations between demographic variables, psychosocial impact, positive and negative experiences with AT and abandonment for hearing aids (**a**) and cochlear implant (**b**). MPT_SOTU+ = Number of positive experiences with technologies; MPT_SOTU− = Number of negative experiences with technologies.

As can be seen in Table 3, overall, lower scores are observed in the PIADS dimensions for the group of people who have abandoned the AT. To explore this relationship in more detail, we first carried out an analysis of the correlations between abandonment and psychosocial impact and other demographic variables (Figure 1).

First, the correlation analysis revealed that abandonment only showed significant and negative correlations with the dimensions of the PIADS scale. In the case of the HA (Figure 1a), the correlations were significant for the three dimensions and denoted a moderate negative relationship (i.e., more abandonment probability for lower values of psychosocial impact) with a higher correlation for the self-esteem dimension (r = −0.63). In the case of CI (Figure 1b), the time with the implant (in years) was also included in the correlational analysis. The only significant correlations with abandonment were for the competence and self-esteem dimensions and, as with hearing aids, the self-esteem dimension showed the highest correlation value (r = −0.77). Secondly, the high correlation between the three dimensions of the PIADS is noteworthy, which seems to denote the possible one-dimensional nature of the psychosocial impact construct. Thirdly, the relationship observed between age and experiences with technologies is noteworthy; thus, the older the age, the fewer positive experiences and the more negative experiences were reported for both groups of AT users.

In order to explore the predictive power of the psychosocial impact and other variables on abandonment for each type of AT, multiple logistic regression analyses were carried out with abandonment (YES/NO) as the dependent variable. A best subset regression procedure [49] was used to select the variables to be included in the models. This procedure aims to find the best fit model from all possible subset models according to goodness-of-fit criteria. In this case, both Bayesian information criterion (BIC) and Akaike information criterion (AIC) were used to build a parsimonious and a complex model respectively.

As can be seen in Table 4, for HA, when AIC was used as the selection criteria, a model with four variables was selected including age, negative experiences with technologies (marginally significant coefficient) and two of the PIADS dimensions, competence and self-esteem. When BIC was used as the criteria, only the competence and self-esteem PIADS dimensions were selected.

Although it was not possible to perform this same analysis on the subsample of CI users, given its small sample size, a similar analysis was performed including users of both HA and CI. As shown in Table 5, when AIC was used as the selection criteria, a model with 3 variables was selected including type of loss (hypoacusis) and two of the PIADS dimensions, competence and self-esteem. When BIC was used as the criteria, only the self-esteem PIADS dimension was selected.

## 4. Discussion

The research carried out for this study aimed to explore the psychosocial impact deriving from the use of different AT and other technologies among deaf and hard of hearing people and the relationship between psychosocial impact and future abandonment of these devices.

First, analyses have shown positive impact results, independent of the type of hearing loss, for all device types. Second, results have also shown that some AT and technologies are associated with better outcomes in terms of their psychosocial impact. For example, it is relevant to note that participants who used VRS, HAs and CIs obtained better outcomes than users of more common ICTs, corroborating the findings of previous studies [14].

Third, the results for hearing aids clearly endorse their use, with a positive psychosocial impact across all types. Previous studies concluded that the use of hearing aids leads to higher satisfaction levels, improved quality of life and greater self-esteem [15,50,51], improved social and emotional wellbeing [52] and greater extroversion and emotional control [42,53,54]. It is relevant to note that no statistically significant differences were found in this study for demographic variables such as gender, age, or type and onset of hearing loss. This echoes the findings of earlier studies, such as that carried out by Solheim, Kværner and Falkenberg [55], which concluded that variables such as age, sex and marital status do not influence limitations on activity or restrictions on participation.

Meanwhile, the results showed a positive impact on quality of life among CI users, corroborating the findings of other studies [56,57,58,59]. Prior research identified improvements in quality of life associated with the use of CI [60,61], as well as improvements in functional capacity. As in other studies, this study also found positive results in older people [62,63].

Finally, the possible relationship between psychosocial impact and abandonment or lack of use of AT was explored. The results for the total PIADS scale and its three subscales showed that the scores obtained by the participants who had abandoned AT a year after the initial study were far lower than those of the other participants (near zero or even negative). Logistic regression analysis were used to supplement these results, pointing to the predictive qualities of the PIADS scale and its three dimensions as useful constructs to determine the probability of future abandonment of an assistive device. Specifically, the results indicate that high competence and self-esteem derived from the use of a hearing aid are associated to a decreased probability of future abandonment of the device. Globally, self-esteem seems to be the dimension most clearly implicated in the abandonment of HA and CI in deaf and hard of hearing people. This result is consistent with those from other studies that have shown the vulnerability of self-esteem in the deaf population and its relation to the satisfaction with different hearing aids and devices [64,65,66]. In the case of competence, some previous studies about hearing aids abandonment have also highlighted the importance of factors that could be related to low levels of competence. For example, McCormack and Fortnum [67] conducted a scoping study to search for reasons for non-use of hearing aids. They found different reasons for non-use like problems of speech clarity, fit and comfort, device factors, or situational factors, among others.

The current study also found a high percentage (20%) of CI non-use or abandonment. However, it is important to emphasize that these results are preliminary and larger-scale studies are required. Although the literature on non-use or abandonment of CI use is scarce, some other studies have provided converging data. For example, Summerfield and Mashall [68] found a 6.3% of non-use between 4–7 years after implantation, which rose to 11% after 7.5 years. In the present study, no statistically significant relationships have been found between abandonment and variables like time since implant, age or other loss-related variables. However, a strong relationship has been verified between abandonment and self-esteem showing the importance of personal factors for device adhesion. Consistently, various studies have shown anxiety, social isolation, stress and depression increases after a CI implantation [63,69]. Indeed, there are several studies that have pointed out the importance of the availability of psychological support for people with CI to address the psychosocial needs and not only the audiological ones [70,71].

Therefore, it seems necessary to carry out longitudinal studies to explore the factors of CI abandonment, considering important dimensions such as Deaf identity, as indicated by other researchers [72], and other psychosocial factors. Qualitative research will also be necessary to explore the factors underpinning the relationship between psychosocial impact and abandonment of auditory devices in greater depth. Overall, we believe that these results are important as research on the abandonment of AT is currently attracting significant interest in the field of outcome measures [73]. Studies such as this are all the more necessary given the stigma attached to hearing loss [31] and the use of certain AT [74,75]. In this respect, the stressful nature of CI surgery should not be overlooked and aspects such as the individual’s opinion, their personal expectations and interests, the communication system chosen, the stigma associated with its use, and other variables related with the changing and improving nature of the devices themselves, like time since implant, must all be taken into account.

A major limitation of this study is the lack of an objective measure of the severity of hearing loss (e.g., audiometry). Although the literature is mixed regarding the relationship between severity of loss and satisfaction with or adoption of a hearing aid, it would have been interesting to explore its possible effect on psychosocial impact. For example, the review of Wong, Hickson and McPherson [76] showed significant relationships (of varied signs) for 5 out of 14 studies, concluding that studies with a larger range of hearing loss are more likely to show a relationship between loss and satisfaction, and in those that did show a relationship, the correlations were low. In other studies, severity of hearing loss, for example hearing sensitivity, has been related with help seeking and uptake but not so clearly to use or satisfaction [77]. Similarly, Kobosko et al. [78] found that CI satisfaction was not related to speech perception scores. But, for example Hosford-Dun and Halpern [79] found a relationship between greater hearing thresholds and a greater amount of use and satisfaction, and Uriarte et al. [80] related higher degrees of hearing loss with higher levels of satisfaction. Thus, a measure of loss severity could have shown a modulating effect on psychosocial impact and for that reason the results should be interpreted with caution.

Furthermore, the non-probability convenience sampling used, as well as the small sample size, complicates the generalizability of the results. Moreover, the abandonment study was only carried out with CI and hearing aid users, so the results cannot be easily generalised to other auditory assistive devices. Also, the factors associated with abandonment have not been systematically explored.

Some other limitations derive from the use of the PIADS scale. First, it is not yet clearly established in the literature that the PIADS subscales can assess separate constructs and this could limit conclusions based on the use of subscale scores. For example, in a recent study analyzing the factor structure of the Spanish version of the scale [81], the results showed an acceptable fit for both single-factor and three-factor correlated solutions. Although more research is needed in this area, we believe that, in any case, the use of the subscales scores could be useful to explore the nature of the difficulties in the adoption of an assistive device, and therefore to propose actions aimed at overcoming those specific difficulties. Second, the PIADS scale is not specific to HA, so it is necessary to carry out studies that compare the different specific and general assessment tools of AT. Furthermore, the PIADS scale evaluates subjective perceptions among participants but it had not been fully validated and adapted for Spanish sign language at the time of the study. This limitation highlights the need for further research to create and validate instruments for evaluating outcome measures that are suited to the needs of people in the Spanish deaf community.

## 5. Conclusions

To conclude, AT can be a useful intervention tool to mitigate the repercussions of hearing loss on people’s daily activities and roles. However, further research is needed on outcome measures for evaluating, selecting and acquiring an AT [35,45,82,83]. The results of this study show that AT for improving hearing has a positive overall psychosocial impact, with improved adaptability, competence and self-esteem observed after beginning their use. Moreover, measures of psychosocial impact can be useful predictors of the future abandonment of a device. Therefore, we believe that results such as those presented in this study are important as they show that low-cost tools, such as outcome measurement instruments, can help to improve processes for evaluating AT, minimising abandonment and lack of use of AT, and promoting the development of more effective devices to meet the needs of final users and improve their day-to-day functioning.

## Figures and Tables

**Table 1:** Socio-demographic profile of deaf and hard of hearing adults using assistive devices or other technologies for communication (n = 291).

Variable	N (%)
Age	M = 56.12; SD = 25.11
Gender	
Male	115 (39.5%)
Female	176 (60.5%)
Language	
Sign language	39 (13.4%)
Spoken language	146 (50.2%)
Both	106 (36.4%)
Residential area	
Rural	60 (20.6%)
Urban	231 (79.4%)
Onset of loss	
Prelocutive	94 (32.3%)
Postlocutive	197 (67.7%)
Type of loss	
Hypoacusis	214 (73.5%)
Cophosis/Total hearing loss	77 (26.5%)

**Table 2:** Overall mean scores (standard deviation) for the Psychosocial Impact of Assistive Devices Scale (PIADS) and the three subscales for each type of assistive device or technology as a function of type of hearing loss.

		PIADS			
	n	Total	Competence	Adaptability	Self-Esteem
Hearing aids (Spectacle, Deep-insertion, In-the-canal, Behind-the-ear) *	137	1.39 (0.93)	1.37 (0.92)	1.41 (0.97)	1.42 (1.09)
hypoacusis	120	1.38 (0.94)	1.35 (.93)	1.39 (0.96)	1.41 (1.02)
cophosis	17	1.47 (0.91)	1.45 (.88)	1.54 (1.09)	1.46 (1.04)
Cochlear implant	30	1.47 (1.13)	1.45 (1.16)	1.47 (0.91)	1.37 (1.28)
hypoacusis	14	1.24 (1.02)	1.17 (1.00)	1.39 (1.11)	1.22 (1.22)
cophosis	16	1.68 (1.21)	1.70 (1.26)	1.88 (1.17)	1.50 (1.40)
Information and Communication Technologies (mobile phone, ooVoo, Skype)	58	0.63 (0.95)	0.59 (0.94)	0.84 (1.18)	0.54 (0.97)
hypoacusis	38	0.57 (0.83)	0.48 (0.79)	0.83 (1.05)	0.49 (0.91)
cophosis	20	0.76 (1.15)	0.80 (1.17)	0.86 (1.43)	0.62 (1.09)
Video Relay Services	66	1.34 (0.87)	1.40 (0.89)	1.61 (1.02)	1.04 (1.02)
hypoacusis	42	1.23 (0.77)	1.38 (0.77)	1.40 (0.96)	0.88 (0.93)
cophosis	24	1.52 (1.01)	1.43 (1.09)	1.98 (1.05)	1.31 (1.12)

* Spectacle: hearing aids integrated into the frames/arms of a pair of glasses/In the canal: These hearing aids fit completely into ear canal and they reach very close to the eardrum./Behind the ear: The main shell section sits behind your ear, and it is connected to a custom-made earmold through a fine clear tube that transmits the amplified sound to the earmold./Deep Insertion (IIC): This kind of hearing aid can be defined by the location of the faceplate, inside the aperture of the ear canal.

**Table 3:** Scores on the PIADS scale and its three subscales according to whether or not participants had abandoned their hearing aid (n = 127) or cochlear implant (n = 30).

		PIADS			
	Abandonment	Total	Competence	Adaptability	Self-Esteem
Hearing aids	NO (n = 102)	1.67 (0.80)	1.62 (0.81)	1.66 (0.88)	1.75 (0.9)
	YES (n = 25)	0.16 (0.25)	0.23 (0.32)	0.27 (0.45)	−0.40 (0.51)
Spectacle	NO (n = 2)	2.77 (0.11)	2.79 (0.18)	2.5 (0.7)	2.94 (0.88)
Deep-insertion	YES (n = 1)	0.42	0.16	0.5	0.75
In-the-canal	NO (n = 29) YES (n = 7)	1.59 (0.78) 0.42 (0.28)	1.55 (0.84) 0.17 (0.3)	1.55 (0.92) 0.50 (0.5)	1.7 (0.84) 0.75 (0.5)
Behind-the-ear	NO (n = 76) YES (n = 17)	1.68 (0.81) 0.11 (0.24)	1.62 (0.81) 0.21 (0.32)	1.7 (0.87) 0.23 (0.44)	1.74 (0.95) −0.13 (0.5)
Cochlear implant	NO (n = 24) YES (n = 6)	1.85 (0.88) −0.03 (0.67)	1.80 (0.96) −0.07(0.79)	1.94 (1.02) 0.47 (0.90)	1.85 (0.89) −0.56 (0.53)

**Table 4:** Logistic regression coefficients (transformed to odds ratio) for two alternative models to predict abandonment of hearing aids, selected with a best regression procedure using Akaike information criterion (AIC) and Bayesian information criterion (BIC) as goodness-of-fit criteria.

Best Model with AIC Criteria			Best Model with BIC Criteria		
	Odds Ratio *	*p*		Odds Ratio	*p*
(Intercept)	0.0004	<0.001	(Intercept)		
Gender (female)	4.22	0.11			
Onset (prelocutive)	0.0003	0.18			
MPT_ SOTU (−)	0.52	0.11			
Competence	0.04	<0.01	Competence	0.12	<0.05
Self-esteem	0.03	<0.01	Self-esteem	0.04	<0.01

* Odds ratio (OR) > 1 indicates increased occurrence of device abandonment; OR < 1 indicates decreased occurrence of device abandonment. MPT_SOTU (−) = Matching Person & Technology, Survey of Technology Use, number of negative experiences with technologies.

**Table 5:** Logistic regression coefficients (transformed to odds ratio) for two alternative models to predict abandonment of hearing aids and cochlear implant, selected with a best regression procedure using AIC and BIC as goodness-of-fit criteria.

Best Model with AIC Criteria			Best Model with BIC Criteria		
	Odds Ratio *	*p*		Odds Ratio	*p*
(Intercept)	0.001	<0.001	(Intercept)	0.009	<0.001
Type of loss (hypoacusis)	4.12	0.15			
Competence	0.27	0.10			
Self-esteem	0.01	<0.001	Self-esteem	0.01	<0.001

* OR > 1 indicates increased occurrence of device abandonment; OR < 1 indicates decreased occurrence of device abandonment.

## Data Availability

The raw data supporting the conclusions of this article will be made available by the authors, on reasonable requests.
